# Prognostic significance of positive peritoneal cytology in endometrial carcinoma confined to the uterus

**DOI:** 10.1038/sj.bjc.6600698

**Published:** 2003-01-28

**Authors:** T Kasamatsu, T Onda, N Katsumata, M Sawada, T Yamada, R Tsunematsu, K Ohmi, Y Sasajima, Y Matsuno

**Affiliations:** 1 Division of Gynecology, National Cancer Center Hospital, 5-1-1 Tsukuji, Chuo-ku, Tokyo 104-0045, Japan; 2 Department of Medical Oncology, National Cancer Center Hospital, 5-1-1 Tsukuji, Chuo-ku, Tokyo 104-0045, Japan; 3 Division of Diagnostic Pathology, National Cancer Center Hospital, 5-1-1 Tsukuji, Chuo-ku, Tokyo 104-0045, Japan

**Keywords:** endometrial carcinoma, peritoneal cytology

## Abstract

A retrospective analysis was performed to evaluate the prognostic significance of peritoneal cytology in patients with endometrial carcinoma limited to the uterus. A total of 280 patients with surgically staged endometrial carcinoma that was histologically confined to the uterus were examined clinicopathologically. The median length of follow-up was 62 (range, 12–135) months. All patients underwent hysterectomy and salpingo-oophorectomy with selective lymphadenectomy, and only three patients received adjuvant postoperative therapy. No preoperative adjuvant therapy was employed. In all, 48 patients (17%) had positive peritoneal cytology. The 5-year survival rate among patients with positive or negative peritoneal cytology was 91 or 95%, respectively, showing no significant difference (log-rank, *P=*0.42). The disease-free survival rate at 36 months was 90% among patients with positive cytology, compared with that of 94% among patients with negative cytology, and the difference was not significant (log-rank, *P=*0.52). Multivariate proportional hazards model revealed only histologic grade to be an independent prognostic factor of survival (*P=*0.0003, 95% CI 3.02 – 40.27) among the factors analysed (age, peritoneal cytology, and depth of myometrial invasion). Multivariate analysis revealed that histologic grade (*P=*0.02, 95% CI 1.21–9.92) was also the only independent prognostic factor of disease-free survival. We concluded that the presence of positive peritoneal cytology is not an independent prognostic factor in patients with endometrial carcinoma confined to the uterus, and adjuvant therapy does not appear to be beneficial in these patients.

Malignant peritoneal cytology is recognised as an adverse prognostic factor in some gynaecologic malignancies. In ovarian cancer, there is a general consensus that postoperative adjuvant chemotherapy should be given to patients with positive peritoneal cytology even if the tumour is limited to the ovaries, that is, the International Federation of Gynecology and Obstetrics (FIGO) stage IC.

As for the positive prognostic value of peritoneal cytology in endometrial carcinoma confined to the uterus, there is still controversy, and conflicting results have appeared in the literature. Accordingly, there is no evidence as to the indication for and efficacy of adjuvant treatment in the case of positive peritoneal cytology. Several studies have reported the prognostic value of positive cytology, and proposed various modalities of adjuvant therapy, that is, multiagent chemotherapy, progestins, whole abdominal radiation, and intraperitoneal radioactive chromic phosphate (^32^P) (McLellan *et al*, 1989; Lurain, 1992). On the other hand, investigators who found that malignant peritoneal cytology has poor prognostic value, found that adjuvant therapy was not beneficial (Yazigi *et al*, 1983; Konski *et al*, 1988; Lurain *et al*, 1989; Kadar *et al*, 1992). The question of the prognostic significance of malignant cytology in endometrial carcinoma confined to the uterus remains unanswered.

This retrospective clinicopathological study was undertaken to identify the prognostic significance of positive peritoneal cytology in endometrial carcinoma confined to the uterus.

## Patients and methods

### Patients

We reviewed the medical records and the cytologic and pathologic materials that had been obtained from 392 patients with surgically treated endometrial carcinoma at the Gynecology Division of the National Cancer Center Hospital, Tokyo, between 1990 and 1998. This study included patients who met the following criteria: the patient underwent primary surgery consisting of total abdominal hysterectomy and salpingo-oophorectomy with selective pelvic and/or para-aortic lymphadenectomy; the patient had no histologic evidence of extrauterine disease; peritoneal cytology was determined in a peritoneal washing obtained by laparotomy immediately upon entering the peritoneal cavity during primary surgery; and the patient had a histologic subtype of endometrioid adenocarcinoma or adenosquamous carcinoma. Patients with uncommon histologic subtypes (mucinous, serous, clear cell, and/or squamous cell carcinoma), and those who had other simultaneous primary malignancy were excluded. All of the patients were surgically staged according to the FIGO staging system (1988), and histologic typing was evaluated according to the criteria of the WHO International Histologic Classification of Tumors.

### Cytopathology

Cytological specimens were obtained by laparotomy upon entering the peritoneal cavity immediately before the primary surgery. Approximately 30 ml of sterile saline was instilled into the pelvis over the uterus, and then aspirated in the cul-de-sac. When a sufficient amount of ascites was present, the fluid was removed with a 20–30-ml syringe. The samples were subjected to cytocentrifugation onto slide glasses at 1700 rpm for 60 s at room temperature. The slides were then fixed in 95% ethanol, followed by Papanicolau stain, and alcian blue stain. Additional slides were stained immunocytochemically for CEA (Mochida, CEA010, Tokyo, Japan), and also for epithelial antigen defined by an antibody BerEP4 (DAKOPATTS, Glostrup, Denmark). Two to three cytotechnologists and cytopathologists independently examined all the slides to make a consensus diagnosis. A patient was considered to have positive peritoneal cytology if adenocarcinoma cells were detected regardless of the number of cancer cells. In this study, in cases where atypical cells were present but could not be definitively identified as cancer cells, the peritoneal cytology was considered to be negative.

### Treatment

Our standard primary treatment for early-stage endometrial carcinoma was surgery consisting of extrafascial total abdominal simple hysterectomy, bilateral salpingo-oophorectomy and selective pelvic and/or para-aortic lymphadenectomy. In cases in which preoperative endometrial biopsy revealed histologic grade 1 tumour and no macroscopic myometrial invasion was found during the operation, lymphadenectomy was not performed. Para-aortic lymphadenectomy was performed if para-aortic node metastasis was diagnosed by pathologic sampling during the operation. Preoperative adjuvant therapy was not employed in any patient, and postoperative adjuvant therapy was not indicated for patients with limited disease.

The primary diagnosis of endometrial carcinoma was made by endometrial biopsy, which had been performed as an office procedure. Hysteroscopy was not performed prior to surgery. Before the surgery, the patients were examined by computed tomography and magnetic resonance imaging. Following the surgery, asymptomatic patients underwent pelvic examination, Pap smear, chest radiograph, ultrasonography, and/or determination of serial tumour markers every 4–6 months. Symptomatic patients underwent the appropriate examination where indicated.

### Statistical methods

Survival and disease-free survival (DFS) curves were obtained by the Kaplan–Meier method and the survival curves were compared by nonparametric survival analysis (log-rank test). Variables that showed a significant association with survival or DFS, and peritoneal cytology were included in multivariate analysis based on the Cox-proportional hazards model. Patients who died of other causes were included as deaths in the survival analysis. Follow-up continued through 30 November, 2001. These statistical analyses were performed using the Statview statistical software package (version 5.0; SAS Institute Inc., Cary, NC, USA).

## Results

### Patient characteristics

In all, 280 patients met the study criteria, with a mean age of 56 years (range, 27–81 years) and a median length of follow-up of 62 months (range, 12–135 months). Of the patients, 112 who underwent surgery for endometrial carcinoma (mean age, 57 years) were excluded. Of these, 46 patients had extrauterine disease including stage III and IV. The remaining patients were excluded because of uncommon histologic subtype, other simultaneous malignancies, and/or inadequate cytologic materials. Of the 280 subjects, 48 patients (17%) had positive peritoneal cytology and 232 (83%) had negative cytology. The characteristics of the patients are summarised in Table 1

**Table 1:** Patient characteristics

	**Positive cytology**	**Negative cytology**
	***n*=48 (%)**	***n*=232 (%)**
*Age (y)*		
Over 60	12 (25)	76 (33)
60 or under	36 (75)	156 (67)
		
*Histologic grade*		
Grade 1	34 (81)	147 (63)
Grade 2	10 (17)	56 (24)
Grade 3	4 (2)	29 (13)
		
*Myometrial invasion*		
Absent	5 (10)	35 (15)
<1/3	20 (42)	106 (46)
1/3–2/3	11 (23)	52 (22)
>2/3	12 (25)	39 (17)
		
*Cervical involvement*		
Absent	34 (70)	198 (85)
Mucosal	7 (15)	6 (3)
Stromal	7 (15)	28 (12)
		
*Lymph – vascular space invasion*		
Absent	34 (71)	172 (74)
Present	14 (29)	60 (26)
		
*Lymph node status*		
Negative	32 (67)	140 (60)
Not resected	16 (33)	92 (40)

. The histologic subtypes were the endometrioid type in 270 cases (96%) and the adenosquamous type in 10 cases (4%). The FIGO stage was as follows: 35 patients (12%) had stage IA disease, 123 (44%) had stage IB, 41 (15%) had stage IC, 5 (2%) had stage IIA, 28 (10%) had stage IIB, and 48 (17%) had stage IIIA. In total, 149 patients (53%) underwent simple hysterectomy and salpingo-oophorectomy with lymphadenectomy; 108 (39%) underwent simple hysterectomy and salpingo-oophorectomy without lymphadenectomy; and 23 (8%) underwent radical hysterectomy. Preoperative radiation therapy, chemotherapy, and progestin therapy were not administered to any patient. Only three patients received postoperative adjuvant therapy. These three patients with stage IIB carcinoma had deep cervical involvement, and external beam radiotherapy to the whole pelvis (total dose of 50 Gy) was administered postoperatively.

### Survival

**Figure 1:**
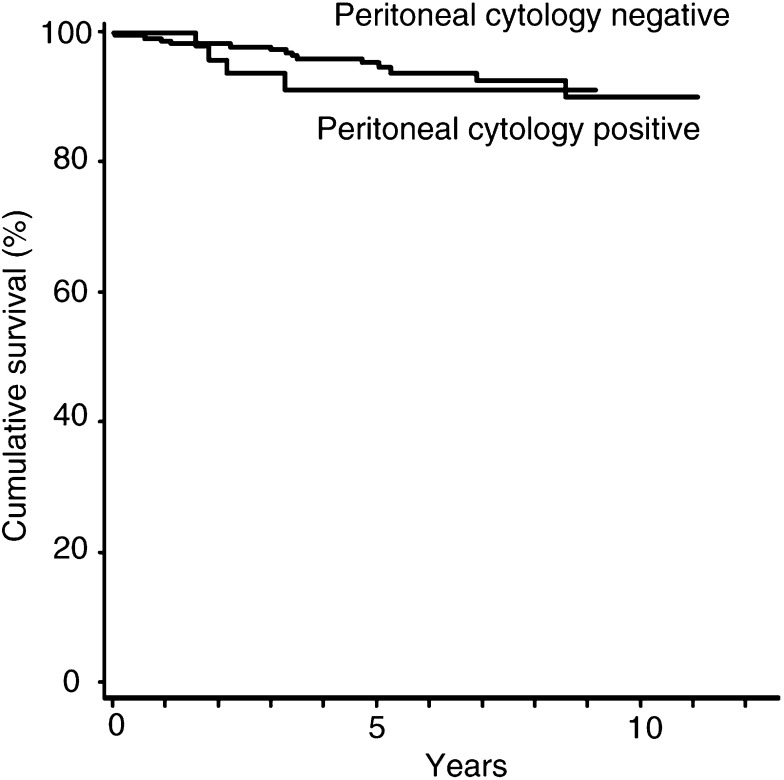
Survival of patients with endometrial carcinoma confined to the uterus according to the presence or absence of malignant peritoneal cytology.

The cumulative survival was assessed in subgroups according to peritoneal cytology (positive or negative), age (over 60 years or 60 years and under), histologic grade (grade 1, grade 2, or grade 3), depth of myometrial invasion (absent, <1/3, 1/3 – 2/3 or >2/3), cervical involvement (absent, mucosal, or stromal), lymph – vascular space invasion (absent or present), and lymph node status (not metastasised or not resected). The 5-year survival rate was 91% among the positive cytology group and 95% among the negative cytology group (Figure 1

). There was no significant difference in survival between patients with positive or negative cytology (log-rank, *P=*0.42). There were no significant differences in the survival of patients in subgroups according to cervical involvement (log-rank, *P=*0.89), lymph– vascular space invasion (log-rank, *P=*0.40), and lymph node status (log-rank, *P=*0.79). Significant differences in survival were found among patients in subgroups according to age, myometrial invasion and histologic grade. Multivariate analysis of testing for differences in survival among the subgroups of cytology, age, depth of myometrial invasion, and histologic grade was performed. The proportional hazards model revealed that only histologic grade was an independent prognostic factor and positive cytology was not an independent adverse prognostic factor ( Table 2

**Table 2:** Univariate analysis and multivariate proportional hazards model for survival

	**Univariate**	**Multivariate**		
	***P-*value**	**Hazard ratio**	**95% CI^a^**	***P*-value**
*Peritoneal cytology*	0.42			
Positive		1.82	0.56–5.86	0.31
*Age (y)*	0.0045			
Over 60		2.50	0.93–6.71	0.06
*Myometrial invasion*	0.02			
<1/3		0.97	0.10–8.66	0.97
1/3–2/3		0.65	0.061–7.07	0.72
>2/3		1.27	0.13–12.35	0.83
*Histologic grade*	<0.0001			
Grade 2		3.28	0.81–13.21	0.09
Grade 3		11.02	3.02–40.27	0.0003

a 95% confidence interval.

).

**Figure 2:**
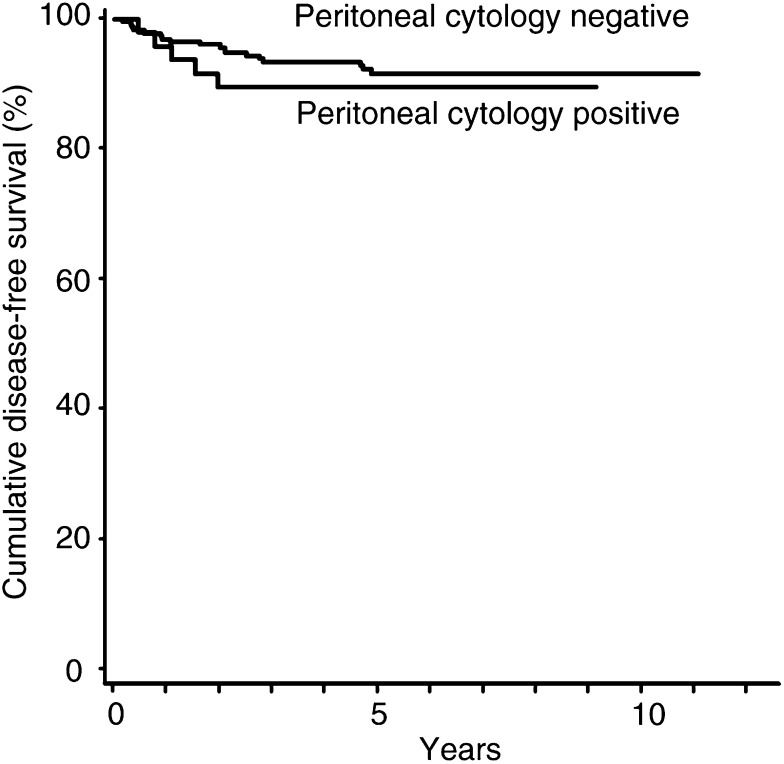
DFS in patients with endometrial carcinoma confined to the uterus according to the presence or absence of malignant peritoneal cytology.

Similarly, the DFS was assessed in the same subgroups. The DFS at 36 months was 90% among the patients with positive cytology, compared with 94% among the patients with negative cytology, and this difference was not significant (log-rank, *P=*0.52) (Figure 2

). Univariate analysis also revealed no significant differences in the DFS of patients in subgroups according to lymph – vascular space invasion (log-rank, *P=*0.29), and lymph node status (log-rank, *P=*0.60). There were significant differences in the DFS of patients in subgroups according to age, myometrial invasion, histologic grade, and cervical involvement. Among these significant subgroups and the subgroup according to peritoneal cytology, the Cox-proportional hazards model showed that only histologic grade was an independent prognostic factor for DFS, and that positive cytology was not an independent factor (Table 3

**Table 3:** Univariate analysis and multivariate proportional hazards model for DFS

	**Univariate**	**Multivariate**		
	***P*-value**	**Hazard ratio**	**95% CI^a^**	***P*-value**
*Peritoneal cytology*	0.52			
Positive		0.83	0.24–2.88	0.77
*Age (y)*	0.005			
Over 60		2.23	0.93–5.32	0.06
*Myometrial invasion*	0.006			
<1/3		1.94	0.23–16.04	0.53
1/3–2/3		2.16	0.23–19.85	0.49
>2/3		3.63	0.39–33.74	0.25
*Histologic grade*	<0.0001			
Grade 2		1.32	0.40–4.30	0.63
Grade 3		3.46	1.21–9.92	0.02
*Cervical involvement*	0.007			
Mucosal		3.47	0.86–14.01	0.07
Stromal		0.55	0.12–2.48	0.44

a 95% confidence interval.

).

### Prognosis and failure sites

Among the 280 patients, 14 patients (5%) suffered tumour recurrence. Table 4

**Table 4:** Clinical characteristics of 14 recurrent patients

**Patient no.**	**Peritoneal cytology**	**Histologic grade**	**Depth of invasion**	**Cervical involvement**	**Initial failure sites**	**Time to recurrence (months)**	**Treatment**	**Status**
1	Positive	1	>2/3	Mucosal	Nodes	24	Not done	DOD^b^ (40)
2	Positive	1	<1/3	Mucosal	Peritoneum	9	Chemo	AWD^c^ (39)
3	Positive	2	<1/3	Absent	Lung	19	Chemo	DOD (22)
4	Positive	3	>2/3	Mucosal	Lung	6	Chemo	DOD (19)
5	Positive	3	>2/3	Absent	Nodes, bone	24	RT^a^	DOD (26)
6	Negative	1	1/3–2/3	Absent	Vagina	4	RT	NED^d^ (116)
7	Negative	1	1/3–2/3	Absent	Vagina	26	RT	NED (64)
8	Negative	1	>2/3	Stromal	Lung, vagina	4	RT, Chemo	NED (72)
9	Negative	1	Absent	Absent	Systemic	26	RT, Chemo	DOD (41)
10	Negative	1	>2/3	Stromal	Lung	13	Surgery	NED (57)
11	Negative	2	>2/3	Absent	Lung	33	Not done	DOD (42)
12	Negative	2	>2/3	Absent	Spleen	24	Surgery	AWD (47)
13	Negative	3	>2/3	Absent	Bone	11	Not done	DOD (13)
14	Negative	3	>2/3	Absent	Lung	31	Unknown	DOD (40)

a Radiation therapy

b Dead of disease

c Alive with disease

d No evidence of disease.

presents the clinical characteristics of the recurrent patients. Peritoneal spread was found in only 20% (one out of five) of the patients with positive cytology who recurred, and the affected site was outside the peritoneal cavity in the remaining 13 patients.

## Discussion

In the past 20 years, over 50 reports on the significance of positive peritoneal cytology in endometrial carcinoma have been published, and many conflicting results have appeared in the literature. Based on studies that found that positive cytology is an independent adverse prognostic factor (Harouny *et al*, 1988; Mazurka *et al*, 1988; Brewington *et al*, 1989; Turner *et al*, 1989; Sutton, 1990; Morrow *et al*, 1991; Grigsby *et al*, 1992; Kadar *et al*, 1994; Descamps *et al*, 1997; Kashimura *et al*, 1997; Obermair *et al*, 2001), postoperative adjuvant therapy was recommended for patients with positive peritoneal cytology. Progestins, whole abdominal external radiation, intraperitoneal radioactive chromic phosphate (^32^P), and multiagent chemotherapy have been proposed. The efficacy of these modalities for treating positive cytology in the absence of other evidence of extrauterine disease is not universally accepted (McLellan *et al*, 1989; Lurain, 1992). On the other hand, investigators who did not find that malignant peritoneal cytology is a significant prognostic factor found no benefit of adjuvant therapy in patients with positive cytology in the absence of other adverse prognostic factors (Yazigi *et al*, 1983; Hernandez *et al*, 1985; Konski *et al*, 1988; Hirai *et al*, 1989; Lurain *et al*, 1989; Grimshaw *et al*, 1990; Kadar *et al*, 1992; Kennedy *et al*, 1993; Ayhan *et al*, 1994; Ebina *et al*, 1997; Yalman *et al*, 2000). This discrepancy is probably because of the following: (1) the reported incidence of positive cytology was approximately 10% and the number of subjects was small; (2) the difference between the surgical stage and the clinical stage was not always distinguished; (3) various modalities of preoperative and/or postoperative therapies were used; (4) in the statistical analysis, multivariate analysis was not always employed; (5) the objectivity of the cytopathologic diagnosis was not always guaranteed; and (6) a prospective study has not been performed.

The prognosis of endometrial carcinoma appears to be good, and an overall 5-year survival rate of 76% can be achieved (Creasman *et al*, 2001) because the majority of patients with endometrial carcinoma have localised, low-grade disease at the time of primary treatment. Indeed, our data indicated that the 5-year survival rate of patients with endometrial carcinoma confined to the uterus was above 90% regardless of positive peritoneal cytology. Additionally, the Cox-proportional hazards model demonstrated that positive peritoneal cytology was not an independent adverse factor for survival and DFS. Although the number of patients in our study was not as large as that in some other studies, all patients were surgically staged and received no preoperative therapy. Only three patients (1%) were treated with postoperative adjuvant therapy. Considering the above facts, it is doubtful whether patients with no extrauterine disease except for positive peritoneal cytology require more aggressive therapy. As for the statistical power, it was difficult to evaluate the power calculation statistically because the number of statistical events was limited and our study was a retrospective one.

In the study of the Gynecologic Oncology Group (GOG) reported by Morrow *et al*(1991), 895 patients with clinical stage I or II (occult) carcinoma of the endometrium were analysed. In total, 29% of the patients with positive cytology developed recurrence compared with 10.5% of the cytology-negative patients, and a relation between malignant cytology and poor outcome was demonstrated by a multivariate model. This GOG study included patients with extrauterine disease, and 42.9% of the patients with no evidence of extrauterine disease received some form of postoperative radiotherapy. Turner *et al*(1989) demonstrated by multivariate analysis that positive cytology was a poor prognostic factor for both the 5-year survival rate (84 *vs* 96%) and progression-free interval (65% at 5 years *vs* 96%) among 567 patients with surgical stage I disease. In that study, 28 women (4.9%) had positive cytology, and the primary treatment was surgery alone for 90 patients (16%), surgery with preoperative adjuvant radiotherapy in 409 patients (72%), and surgery with postoperative adjuvant radiotherapy in 46 patients (8%). Preoperative radiotherapy may have affected the surgical stage and peritoneal cytology of many patients enrolled in that study.

Similarly, in many previous studies that found that positive peritoneal cytology had no prognostic significance, we found the same problems; for example, many patients received pre- or postoperative adjuvant therapy, or multivariate analysis was not employed. Grimshaw *et al*(1990) showed that there was no significant difference in the 5-year survival rate between patients with positive or negative cytology (80 *vs* 86%) among 305 surgical stage I patients. In that study, statistical significance was analysed with only the Fisher exact test. Kadar *et al*(1992) demonstrated that positive cytology did not influence survival if the disease was confined to the uterus using Cox's proportional hazards model. In that study, treatment variables included the use of adjunctive radiation therapy and the type of radiation therapy used, and 59% (159 out of 269) of the patients received radiation therapy. In the present study, no patient received preoperative therapy and only three (1%) of the 280 patients received postoperative adjuvant therapy.

Positive cytology was not an adverse prognostic factor in endometrial carcinoma limited to the uterus, and it is unknown from where these cancer cells were derived. Although there are insufficient data to reach a conclusion about the source of the cancer cells in peritoneal washings, the following mechanisms may be deduced from the literature (McLellan *et al*, 1989; Lurain, 1992): (1) result of transtubal transport; (2) direct extension of tumour through the myometrium; (3) lymphatic metastasis to the peritoneal cavity; and (4) reflection of multifocal peritoneal occult spread. The transtubal transport theory seems to be the most popular. Hirai *et al*(2001) demonstrated by using a tube that was inserted into the abdomen during the operation for cytologic analysis, that positive peritoneal cytology usually disappeared within a short period of time after the operation (within 14 days) in patients with limited disease in comparison to patients with adnexal metastasis. Additionally, as for the failure site in the present series, peritoneal spread was found in only 20% of the patients with positive cytology who recurred, and in the remaining patients, the affected site was outside the peritoneal cavity. Another study (Lurain *et al*, 1989) showed that 17% of patients with stage I disease who had positive cytology suffered recurrence, and only 20% of these recurrences were within the abdomen. The above-mentioned findings suggest that malignant cells obtained by peritoneal washing may not reflect the potential of peritoneal spread in a significant proportion of endometrial carcinoma cases unless other extrauterine disease is present.

In most studies including the present study, peritoneal cytology was analysed by conventional cytopathologic techniques and morphologic findings. Although cytopathologic findings including adequate sampling are essential for analysing the prognostic value of peritoneal cytology, evaluating the objectivity of cytopathologic diagnosis is difficult. The available data indicated that among 3091 reported cases with clinical stage I disease, the overall incidence of positive cytology was 11.4% (range, 2.9–29.8%) (McLellan *et al*, 1989). If the positive rate in a study is rather high, the possibility that reactive mesothelial cells were confused with malignant cells must be considered. If the positive rate in a study is too low, sampling error should be considered. Szpak *et al*(1981) demonstrated that the presence of abundant malignant cells (greater than 1000 cells per 100 ml sample) significantly shortened the time to recurrence. Yanoh *et al*(1999) proposed that the finding**s** of endometrial adenocarcinoma cells exhibiting high cellularity, scalloped edge of cell clusters and isolated cells in peritoneal cytology could be regarded as a risk factor for intra-abdominal recurrence. Luo *et al*(2001) reported that analysis of peritoneal washings with conventional and immunocytochemical (MOC-31) staining improved the diagnosis of peritoneal cytology in endometrial carcinoma, and positive combined cytology was a prognostic factor. The results of research on these morphological findings have not yet been widely accepted, and will be worthy of consideration in the future.

Currently, we believe that the presence of positive peritoneal cytology is not an independent prognostic factor, and that it does not seem to reflect the potential of peritoneal spread in patients with endometrial carcinoma confined to the uterus. Adjuvant therapy such as chemotherapy, radiation therapy, or progestins does not appear to be beneficial in these patients at present. Nonetheless, further investigation and prospective multiinstitutional prospective analyses are needed.
